# Survival control of oligodendrocyte progenitor cells requires the transcription factor 4 during olfactory bulb development

**DOI:** 10.1038/s41419-020-03371-3

**Published:** 2021-01-18

**Authors:** Yilan Zhang, Yuqun Cai, Yafei Wang, Xin Deng, Yifan Zhao, Yubin Zhang, Yunli Xie

**Affiliations:** 1 Department of Anesthesia, State Key Laboratory of Medical Neurobiology and MOE Frontiers Center for Brain Science, Institutes of Brain Science, Zhongshan Hospital, Fudan University, Shanghai, 200032 China; 2 School of Public Health/MOE Key Laboratory for Public Health Safety, Fudan University, Shanghai, 200032 China

**Keywords:** Developmental neurogenesis, Glial development

## Abstract

A proper number of oligodendrocytes in the nerve system is essential for neuronal functions. In the olfactory bulb (OB), enriched oligodendrocytes are crucial for olfactory information processing. However, how the precise number of oligodendrocytes in the OB is regulated remains elusive. Here we identified that the transcription factor 4 (Tcf4)-mediated cell death is essential for generating an appropriate number of oligodendrocyte progenitor cells (OPCs) and thereby oligodendrocytes in the OB. We showed that Nkx2.1-positive progenitors in the medial ganglionic eminence (MGE) and anterior entopeduncular area (AEP) provide the first source of OPCs in the OB. Conditional depletion of Tcf4 leads to an increase of OPCs in the OB, which is mediated by the suppression of programmed cell death. Furthermore, we showed that Tcf4 mediated OPC survival is cell-autonomous by transplantation assay. Mechanistically, we identified Bax/Bak as a potential key pathway to promote OPC elimination during OB development. Depletion of Bax/Bak in Nkx2.1 lineage results in an increase of OPCs in the OB. Mutations in TCF4 causes Pitt-Hopkins syndrome, a severe neurodevelopmental disorder. Thus, our findings reveal an important intrinsic mechanism underlying the survival control of OPCs in the OB and provide new insights into the pathogenesis of Pitt–Hopkins syndrome.

## Introduction

A precise number of oligodendrocytes is essential for neuronal functions through the myelination of axons in the central nervous system (CNS)^1–3^. Oligodendrocytes develop from oligodendrocyte precursor cells (OPCs), which originate from the radial glial cells (RGCs) of the ventricular zone in several regions of the embryonic brain^4^. In contrast to mature oligodendrocytes, OPCs maintain their proliferative capacity and continue to differentiate into oligodendrocytes in the adult brain^5^. However, OPCs derived from distinct origins are functionally redundant and populate the cortex in sequential waves^6,7^, suggesting that the number of OPCs produced from distinct origins is tightly controlled. Programmed cell death is one of the key mechanisms in controlling the precise number of cells, including neurons and glial cells by eliminating the excess during brain development^8–11^.

RGCs expressing the homeodomain transcription factor Nkx2.1 in the ventricular zone of the MGE and AEP generates most of the interneurons in the cortex^12^. After differentiation, these interneurons migrate tangentially to the dorsal cortex to form connections with local projection neurons^13–15^. In addition to the production of interneurons, Nkx2.1-positive RGCs also contribute to the generation of the first wave of OPCs which further differentiate into mature oligodendrocytes in the neocortex^6,16^. While extensive studies have focused on how OPCs are generated from Nkx2.1-positive RGCs in the neocortex, the contribution of these RGCs to the production of OPCs in the OB, the essential structure involved in olfaction, is largely unknown.

Tcf4 (also known as E2-2 and ITF2), a basic helix–loop–helix (bHLH) transcription factor, is highly expressed in both developing and adult brains^17–19^. TCF4 mutations cause Pitt–Hopkins syndrome, a neurodevelopmental disorder characterized by developmental delay and intellectual disability and is highly associated with schizophrenia^20,21^. Tcf4 plays essential roles in hippocampal development and cortical lamination in the developing brain^22–24^. Perturbations of *Tcf4* in mouse models disrupt synaptic function which leads to deficits in learning and memory^25–27^. The expression of Tcf4 has been detected in various cell types, including neurons, astrocytes, and oligodendrocytes^18^. However, the role of Tcf4 in regulating oligodendrocyte development remains largely unknown. Here, we conditionally inactivated Tcf4 in the Nkx2.1 lineage and showed that Tcf4 plays an essential role in regulating OPC survival during OB development.

## Results

### Nkx2.1-positive RGCs generate OPCs in early OB developmental stages

**Fig. 1:**
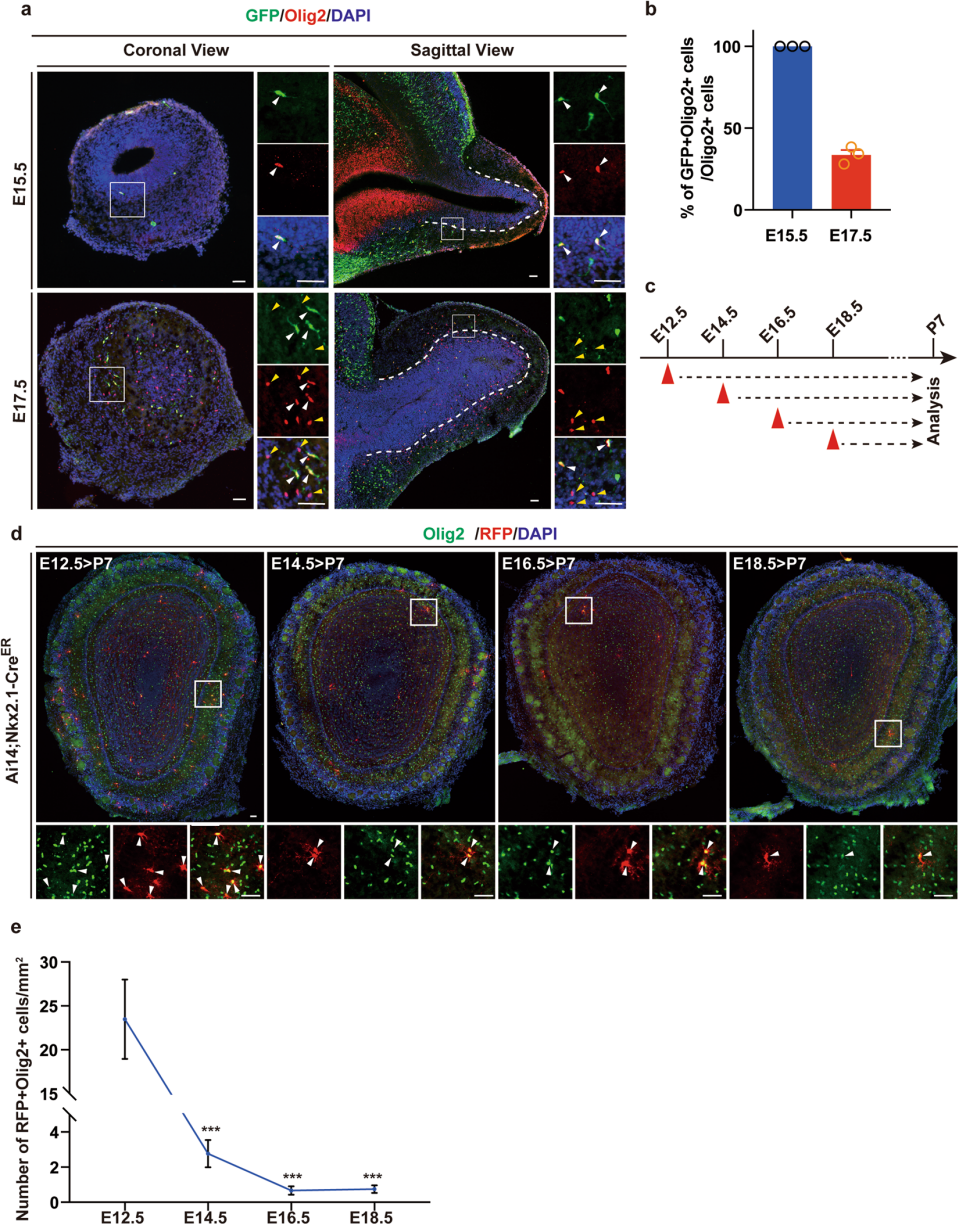
Nkx2.1 lineage generates OPCs in the OB at early stages. **a** Coronal and sagittal sections of the OB from Ai3;Nkx2.1Cre mice stained for GFP and Olig2 at E15.5 and E17.5. White arrowheads indicate Nkx2.1-positive RGCs derived OPC lineage cells (GFP+Olig2+). Yellow arrowheads show other source derived OPC lineage cells (Olig2+GFP−). The dashed lines represent the boundary of the olfactory ventricle zone and the granule cell layer. The small mono-channels are the higher magnification of the solid square boxes. Scale bar: 50 μm. **b** Quantification of Nkx2.1-positive RGCs derived Olig2-positive cells at E15.5 and E17.5. Data are presented as mean ± SEM. At least 3 mice were analyzed for each time point. **c** Experimental paradigm shows that tamoxifen (TAM) was injected at E12.5, E14.5, E16.5, and E18.5 and sacrificed at P7. **d** Confocal images of sections of the OB from Ai14;Nkx2.1-Cre^ER^ mice stained for Olig2 and RFP. Arrowheads indicate the RFP- and Olig2-double-positive cells. Scale bar: 50 μm. **e** Quantification of the number of Nkx2.1-derived OPC lineage cells generated at E12.5, E14.5, E16.5, and E18.5. One-way ANOVA, data are presented as mean ± SEM. ***p < 0.001 (*n* = 3).

To examine how Nkx2.1-positive RGCs generate OPCs of the OB, we first crossed Nkx2.1Cre mice with Ai3 reporter mice, which allows us to label Nkx2.1-positive progenitors of the MGE and AEP, and thereby their progenies starting from the early stage of cortical development around embryonic day 10.5 (E10.5)^14^. At E15.5, we observed a small number of GFP-positive cells in the OB (Fig. 1a). Remarkably, when stained the sections for Olig2, a marker for OPCs and mature oligodendrocytes, we found that all Olig2-positive cells were positive for GFP (Fig. 1a, b), suggesting that Nkx2.1-positive RGCs generate the first wave of OPCs in the OB. At E17.5, we observed that around 30% of Olig2-positive cells were expressing GFP (Fig. 1a, b). To further examine the time course of Nkx2.1-positive RGCs generating OPCs in the OB, we crossed Ai14 reporter mice with Nkx2.1Cre^ER^ mice and induced the Cre expression through tamoxifen induction at different developmental stages (Fig. 1c). When analyzed the OB at postnatal day 7 (P7), we found that most OPCs were derived from RGCs labeled at E12.5 (Fig. 1d, e). Taken together, these data demonstrated that Nkx2.1 lineage contributed to the generation of early OPCs in the OB.

### Depletion of Tcf4 leads to an increase of OPCs in the OB

**Fig. 2:**
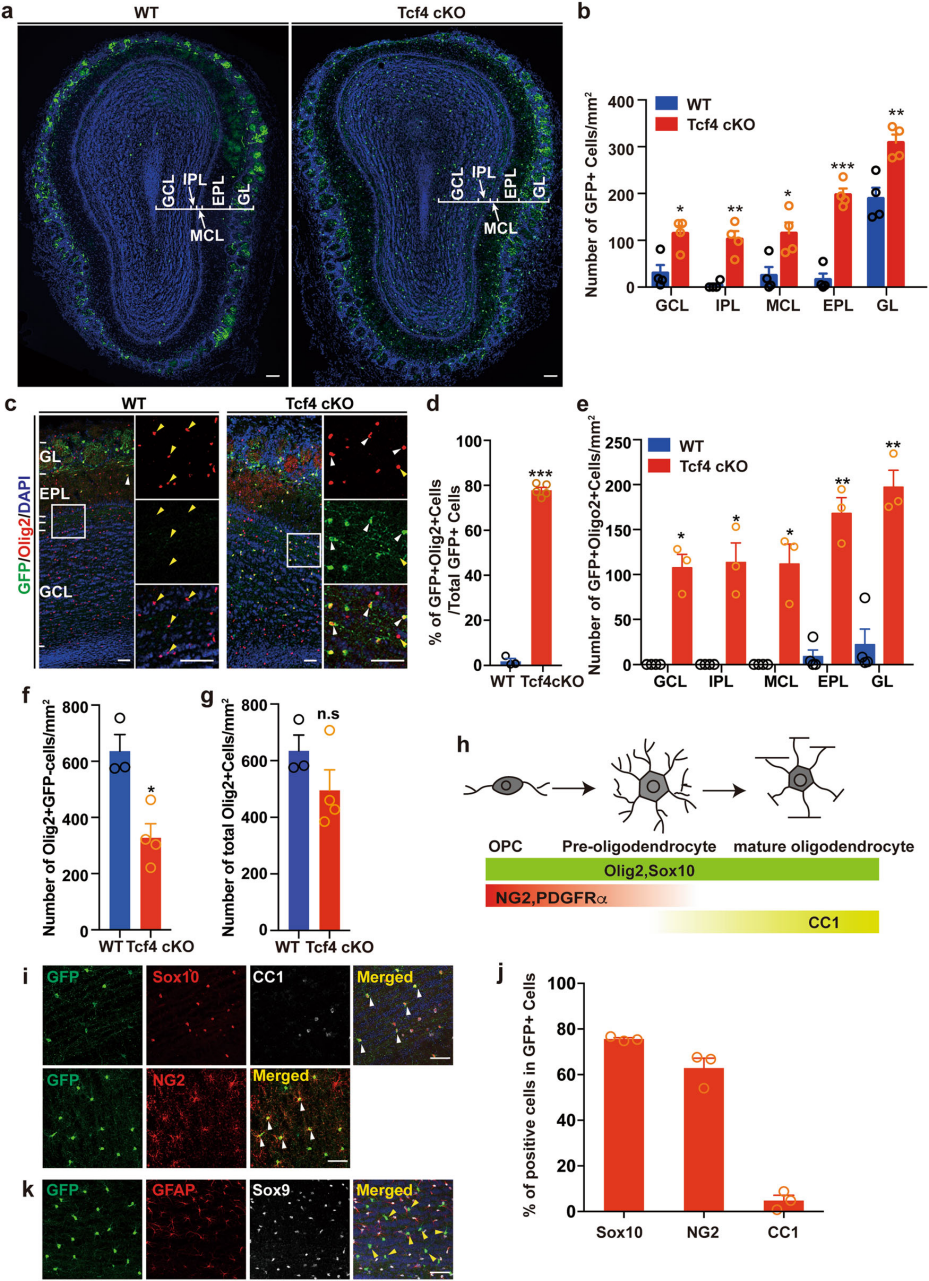
Conditional deletion of Tcf4 in Nkx2.1 lineage leads to an increase of OPCs in the OB. **a** Representative images showing GFP-positive cells from WT and Tcfk4 cKO OB. Scale bar: 100 μm. **b** Quantification of the distribution of GFP-positive cells. Student’s *t* test was used to compare two groups. Data are presented as mean ± SEM. *p < 0.05, **p < 0.01, and ***p < 0.001 (*n* = 4). **c** Representative images of RFP and Olig2 staining in WT and Tcf4 cKO OBs at P28. Small monochannels are the higher magnification of the solid square boxes. White arrowheads represent the GFP+Olig2+ cells and yellow arrowheads mark GFP−Olig2+ cells. Scale bar: 50 μm. **d**–**g** Quantifications of Nkx2.1-derived Olig2-positive cells (**d**) and their distribution in each layer of the OB (**e**), the number of non-Nkx2.1 derived Olig2-positive cells (**f**), and the total number of Olig2-positive cells in the OB (**g**). Student’s *t* test was used to compare two groups. Data are presented as mean ± SEM. *p < 0.05, **p < 0.01, n.s. nonsignificant. At least three samples were analyzed for each condition. **h** Diagram shows molecular markers expressed during OPC development. **i** Representative images show the immunostaining for GFP, Sox10, CC1, and NG2. White arrowheads indicate GFP+Sox10+ cells negative for CC1. **j** Quantification of cells is positive for either Sox10 or NG2 or CC1. **k** Representative images show the immunostaining for GFP, GFAP, and Sox9. Yellow arrowheads indicate GFP-positive cells which are negative for GFAP and Sox9. Scale bar: 50 μm.

By performing in situ hybridization, we found that the expression of *Tcf4* was highly abundant in the ventral cortical regions, such as MEG and AEP (Fig. S1). In addition, it has been reported that *Tcf4* is expressed in OPCs by analyzing RNA sequencing data although its role in OPC development has not been addressed^28^. To ask whether Tcf4 regulates OPC development, we conditionally inactivated Tcf4 in Nkx2.1-lineage specifically by crossing Tcf4^f/f^ mice with Nkx2.1Cre line to generate Tcf4 conditional knockout mice (Tcf4^f/f^;Nkx2.1Cre, Tcf4 cKO). To visualize the Nkx2.1 progeny, we crossed either Tcf4 cKO or WT (Nkx2.1Cre) mice with Ai3 or Ai14 reporter mice^29^. When analyzed at P28, we found a significant increase of GFP-positive cells in the OB of Tcf4 cKO compared to that of WT (Fig. 2a, b). As these cells were different from neurons and astrocytes morphologically, we reasoned that these cells might be oligodendrocytes. Indeed, when stained the sections of both WT and Tcf4 cKO for markers expressed in the oligodendrocyte lineage (Fig. 2c), we found that nearly 80% of GFP-positive cells were labeled by Olig2 in the OB of Tcf4 cKO at P28, while very few Olig2- and GFP-double positive cells in the OB of WT were detected (Fig. 2d). The distribution of these Olig2-positive cells was found in all laminar layers in the OB of Tcf4 cKO (Fig. 2e). Interestingly, when analyzed the number of Olig2-positive and GFP-negative cells which were originated from another source, we found a reduction of this population in the OB of Tcf4 cKO (Fig. 2f), suggesting a compensation mechanism that maintains oligodendrocyte homeostasis. Indeed, the total number of Olig2-positive cells was not altered upon the loss of Tcf4 (Fig. 2g). The expression of Olig2 in the oligodendrocyte lineage begins from the OPC stage and persists to the mature stage (Fig. 2h)^30^. Similarly, Sox10 is also expressed in different developmental stages during oligodendrocyte maturation (Fig. 2h)^5^. The increase of oligodendrocytes in the OB of Tcf4 cKO was further confirmed by the staining of Sox10 (Fig. 2i). We then asked whether the increased Olig2-positive cells were OPCs or mature oligodendrocytes (Fig. 2h) and stained for NG2, a marker for OPC^31^. We found that most cells were positive for NG2 and not positive for mature oligodendrocyte marker CC1 (Fig. 2i, j), suggesting that they are OPCs. Even in the aged adult mouse OB, they were positive for NG2, but not CC1 (Fig. 2S). We also confirmed that these cells were not astrocytes as they were negative for GFAP and Sox9, markers for astrocytes (Fig. 2k). Collectively, these data demonstrated that loss of Tcf4 led to the increase of OPCs in the OB.

### Differentiation of neural progenitor cells remains unaffected upon the loss of Tcf4

**Fig. 3:**
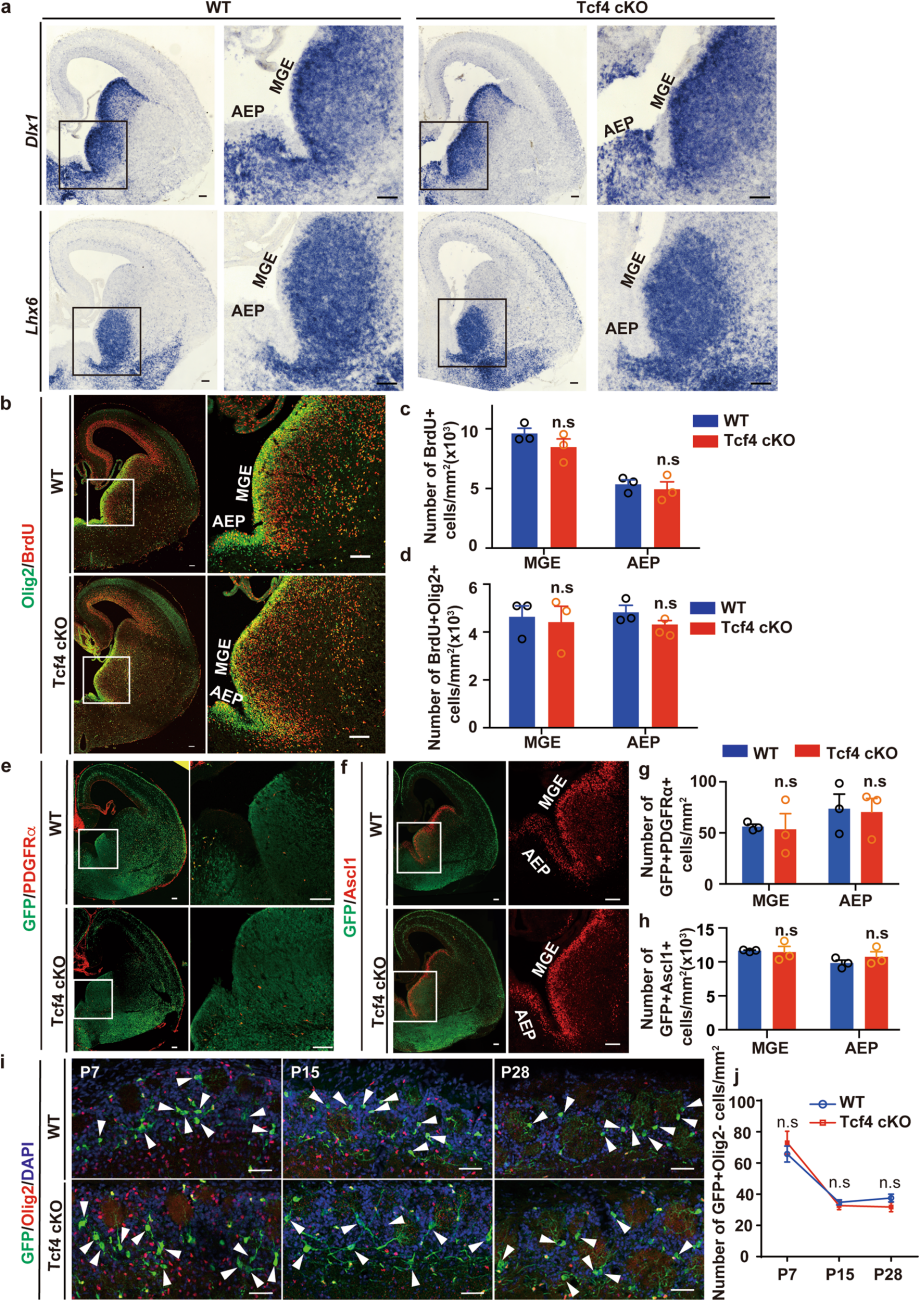
Proliferation and differentiation of Nkx2.1-RGCs were not altered upon the loss of Tcf4. **a** In situ hybridization with *Dlx1*and *Lhx6* probes from WT and Tcf4 cKO mice at E15.5. Higher magnification images of the inserts showed on the right panels. Scale bar: 100 μm. **b** Representative images of immunostaining for GFP, Olig2, and BrdU. The right panel shows the higher magnification of the inserts. Scale bar: 100 μm. **c**, **d** Quantification of the proliferating progenitors (**c**) and proliferating OPCs (**d**). Student’s *t* test was used to compare two groups. Data are presented as mean ± SEM. n.s. nonsignificant. **e**, **f** Immunostaining for PDGFRα (**e**) and Ascl1 (**f**). Scale bar: 100 μm. **g**, **h** Quantification of PDGFRα-positive (**g**) and Ascl1-positive (**h**) progenitors. Student’s *t* test was used to compare two groups. Data are presented as mean ± SEM. n.s. nonsignificant. **i** Representative images show immunostaining for GFP and Olig2 at P7, P15, and P28 from both WT and Tcf4 cKO OBs. Neurons are negative for Olig2 and display distinct morphology (arrowheads). Scale bar: 50 μm. **j** Quantification of neurons at different developmental stages of OB as indicated. Student’s *t* test was used to compare two groups. Data are presented as mean ± SEM. n.s. nonsignificant.

To ask whether the increased OPCs was due to the decreased differentiation of RGCs toward the neuronal lineage upon the loss of Tcf4 in the MGE and AEP, we first examined the expression of *Dlx1* and *Lhx6*, markers for differentiated neural progenitors, by performing the in situ hybridization (Fig. 3a). We found that the expression of *Dlx1* and *Lhx6* was not altered in the MGE and AEP of Tcf4 cKO compared to these of WT (Fig. 3a). To examine the proliferation capacity of RGCs in the MGE and AEP, we administrated bromodeoxyuridine (BrdU) to mouse embryos at E15.5 and then analyzed them 2 h later. We found that the number of BrdU-positive progenitor cells was not altered in Tcf4 cKO compared to WT (Fig. 3b, c). Particularly, the proliferation of OPCs remained unchanged upon the loss of Tcf4 (Fig. 3b, d). This was further confirmed by staining the sections for PDGFRα and Ascl1, markers for differentiated OPCs^32^ (Fig. 3e–h). Therefore, the increased OPCs in the OB of Tcf4 cKO did not result from the increased differentiation of RGCs toward the oligodendrocyte lineage. Nkx2.1-positive RGCs of MGE and AEP not only generate oligodendrocytes but also interneurons of the OB located at the glomerular layer of the OB with distinct morphology^14,33^. We then asked whether the number of interneurons was also altered in the OB of Tcf4 cKO. As no specific markers to label interneurons of the OB derived from Nkx2.1-positive RGCs^14,33^, we quantified the number of cells positive for GFP but negative for Olig2. At P7, we found that the number of interneurons in the OB of Tcf4 cKO was comparable to that of WT (Fig. 3i, j). However, the number of interneurons was declined at P15 and P28 in the OB of both WT and Tcf4 cKO (Fig. 3j). Taken together, our results showed that the increased OPCs in the OB of Tcf4 cKO was not due to the increase of neural progenitor differentiation toward oligodendrocyte lineage.

### Tcf4 promotes OPC programmed cell death during OB development

**Fig. 4:**
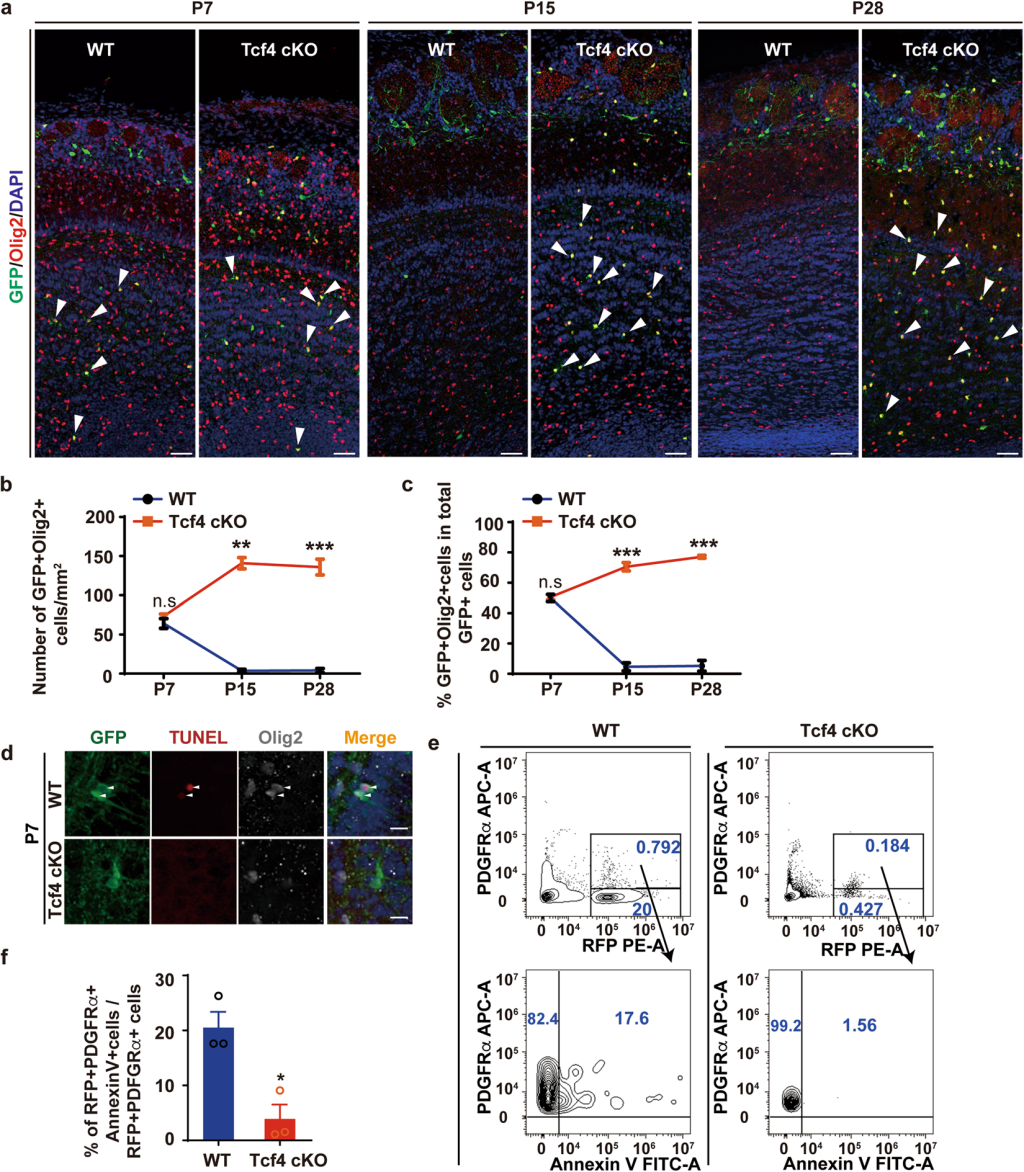
Loss of Tcf4 results in programmed cell death in OPCs. **a** Representative images of immunostaining for GFP and Olig2 at various developmental stages in both WT and Tcf4 cKO OBs. Arrowheads show OPCs. Scale bar: 50 μm. **b**, **c** Quantification of the density of OPCs (**b**) and the proportion of Nkx2.1-derived OPCs (**c**). Student’s *t* test was used to compare two groups. Data are presented as mean ± SEM. *p < 0.05, **p < 0.01, and ***p < 0.001. At least three OBs were analyzed for each genotype. **d** Representative images of immunostaining of GFP, TUNEL, and Olig2 in WT and Tcf4 cKO OBs at P7. Arrowheads indicate the OPCs undergoing cell death. Scale bar: 25 μm. **e** FACS analysis of OPCs undergoing cell death. **f** Quantification of the proportion of AnnexinV-positive OPCs in both WT and Tcf4 cKO OBs. Student’s *t* test was used to compare two groups. Data are presented as mean ± SEM. *p < 0.05, **p < 0.01, and ***p < 0.001. At least three OBs were analyzed for each genotype.

Another possibility for the increase of OPCs in the OB of Tcf4 cKO is the reduction of OPC apoptosis. Due to the transient expression of the classical apoptotic markers and quick clearance of dying cells, it is difficult to directly quantify the dying cells by immunostaining for apoptotic markers^6,10^. Therefore, we first examined the number of OPCs at different developmental stages (Fig. 4a). At P7, we found that the number of OPCs in the OB was comparable between WT and Tcf4 cKO (Fig. 4a, b). However, in the WT OB, the number of OPCs declined after the first postnatal week and became nearly undetectable at P15 and at P28 (Fig. 4a, b). In contrast, the number of OPCs increased after P7 and persisted till adulthood at P28 in Tcf4 cKO OB (Fig. 4a–c), indicating a reduction of the OPC apoptosis in the lack of Tcf4. Indeed, we detected more DNA breaks, formed in the last phase of apoptosis, in the OB of WT compared to that of Tcf4 cKO by performing TUNEL assay, although the number is low due to the quick clearance most likely (Fig. 4d)^10^. To further confirm the reduction of the cell death when Tcf4 is depleted, we sorted OPCs from the OB of both WT and Tcf4 cKO by fluorescence activating cell sorting using the surface marker PDGFRα while examining the expression of Annexin V, a surface marker for cells undergoing apoptosis^34^ (Fig. 4e). Indeed, we found that the number of OPCs expressing Annexin V was significantly decreased in the OB of Tcf4 cKO (Fig. 4f). Taken together, these data demonstrated that OPCs in the WT OB underwent apoptosis after the first postnatal week and were nearly undetectable in adulthood.

### Tcf4 regulates the survival of OPCs cell-autonomously

**Fig. 5:**
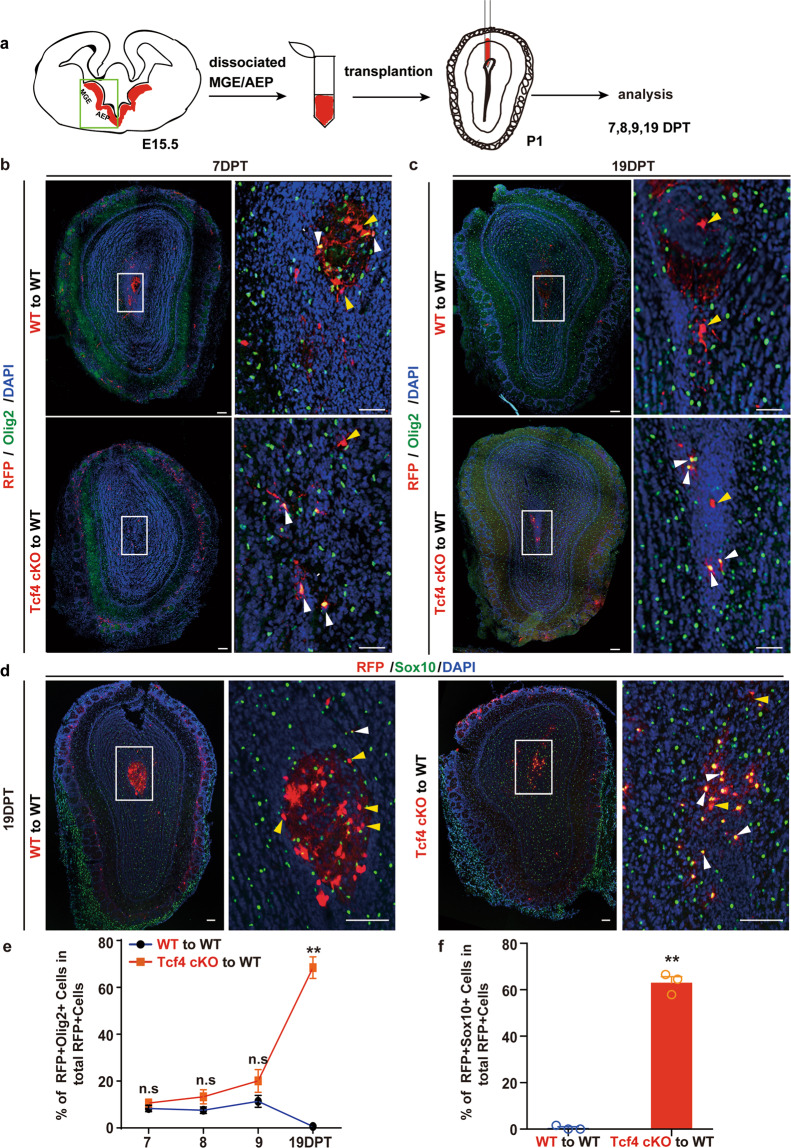
Tcf4 regulates OPC survival cell-autonomously. **a** Schematic diagram illustrating the transplantation experiment. **b**–**d** Representative images showing that the WT or Tcf4 cKO progenitors of MGE differentiate into OPCs 7DPT (**b**), and OPCs lacking Tcf4 survived 19DPT (**c**). The survived OPCs expressing Sox10 (**d**), an OPC marker. The enlarged images were shown on the right panels. Yellow arrowheads indicate Olig2-negative cells and white arrowheads indicate OPCs. Scale bar: 100 μm. **e**, **f** Quantifications of OPCs after transplantation (**e**) and the number of Sox10-positive OPCs (**f**). Student’s *t* test was used to compare two groups. Data are presented as mean ± SEM. **p < 0.01, n.s. nonsignificant. At least three OBs were analyzed for each genotype at each time point.

To further examine whether Tcf4 acts cell-autonomously or non-autonomously to regulate OPC survival, we performed transplantation experiments (Fig. 5). In this assay, we dissociated RGCs of MGE and AEP from either control (Ai14;Nkx2.1Cre) or Tcf4 cKO (Tcf4^f/f^;Ai14;Nkx2.1Cre) embryos at E15.5 and then transplanted these cells into the OB of the WT mice at P1 (Fig. 5a). Transplanted animals were analyzed at various days post-transplantation (DPT). After transplantation, RGCs differentiated into OPCs and neurons which were visualized by RFP reporter. We stained sections for Olig2 to examined the number of OPCs. When analyzed at 7 DPT, the number of OPCs of both WT and Tcf4 cKO OBs was similar (Fig. 5b, e). At 19 DPT, however, we hardly observed Olig2-positive OPCs when WT progenitor cells were transplanted (Fig. 5c, e), suggesting that WT OPCs underwent apoptosis as endogenous WT OPCs did. Interestingly, we observed that OPCs survived when progenitor cells lacking Tcf4 were transplanted into the WT OB (Fig. 5c, e). The identity of OPCs was also confirmed by the staining for Sox10, another marker for OPCs (Fig. 5d, f). A series of analysis at different time points after the transplantation showed that WT OPCs persisted in the host OB till 9 DPT and then underwent apoptosis while OPCs lacking Tcf4 survived till 19 DPT (Fig. 5e), which is similar to what we observed for endogenous OPCs. Thus, these data showed that Tcf4 regulated OPCs survival in a cell-autonomous manner.

### Increased proliferation of survived OPCs lacking Tcf4

**Fig. 6:**
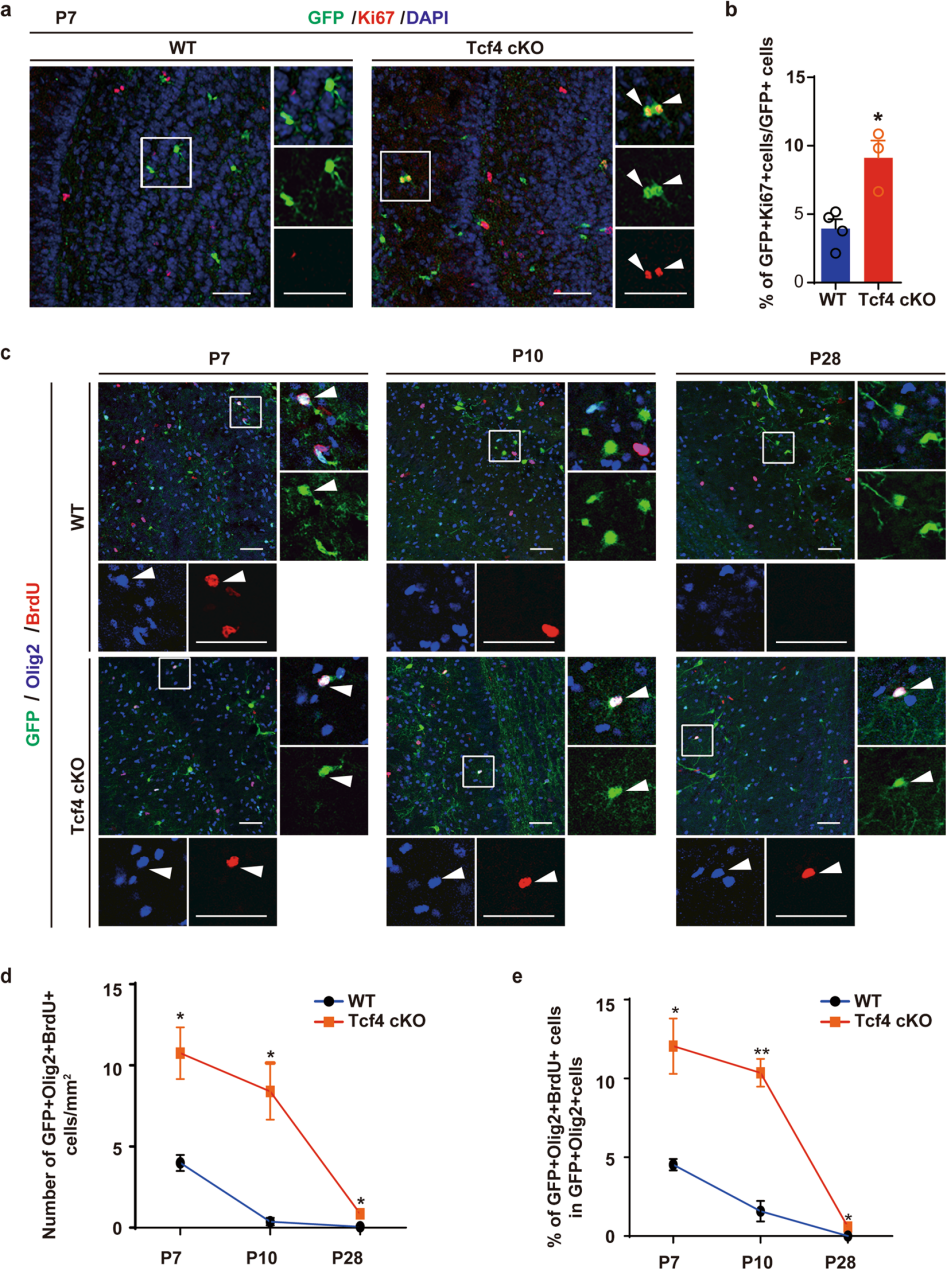
Proliferation of OPCs in Tcf4 cKO OB. **a** Sections of OB from WT and Tcf4 cKO stained for GFP and Ki67 at P7. Scale bar: 50 μm. **b** Quantification of proliferating OPCs in both WT and Tcf4 cKO OBs. Student’s *t* test was used to compare two groups. Data are presented as mean ± SEM. *p < 0.05. At least three samples were analyzed for each genotype. **c** Representative images show sections of OB from WT and Tcf4 cKO stained for Olig2 and BrdU at P7, P10, and P28. Arrowheads indicate proliferating OPCs which are BrdU-positive. Scale bar: 50 μm. **d**, **e** Quantification of the density of proliferating OPCs (**d**) and the number of proliferating OPCs derived from the Nkx2.1 lineage (**e**). Student’s *t* test was used to compare two groups. Data are presented as mean ± SEM. *p < 0.05, **p < 0.01. At least three OBs were analyzed for each genotype at each time point.

We observed that the number of OPCs in the Tcf4 cKO OB increased gradually from P7 to P28 (Fig. 4c). Likely, this was due to the proliferation of survived OPCs in the Tcf4 cKO OB as OPCs are known to proliferate till adulthood^5^. To test this hypothesis, we first stained the sections for Ki67, a marker for cell proliferation. We found that most progenies derived from Nkx2.1-positive RGCs were not positive for Ki67 in the WT OB (Fig. 6a). However, we found many pairs of Ki67-positive cells in the Tcf4 cKO OB, suggesting they were proliferating (Fig. 6a, b). To further examine the proliferative status of these OPCs, we administrated BrdU into the postnatal mice and then analyzed 2 h later. We found that some of the Olig2-positive OPCs of both WT and Tcf4 cKO were positive for BrdU at P7 (Fig. 6c), although the number of proliferating OPCs in the WT OB was significantly lower than in the Tcf4 cKO OB (Fig. 6c–e). As OPCs underwent apoptosis in the WT OB after the first postnatal week, the number of OPCs positive for BrdU was dramatically reduced at P10 and P28 (Fig. 6d, e). In contrast, many OPCs that survived in the Tcf4 cKO OB were positive for BrdU even after P7 (Fig. 6c–e), indicating that these survived OPCs were capable of proliferation. However, the number of proliferating OPCs in the Tcf4 cKO OB declined during OB development (Fig. 6e). Therefore, the persisted proliferation of survived OPCs in the Tcf4 cKO OB may also contribute to the increased number of OPCs.

### Tcf4 acts through Bax/Bak pathway to promote the cell death of OPCs

**Fig. 7:**
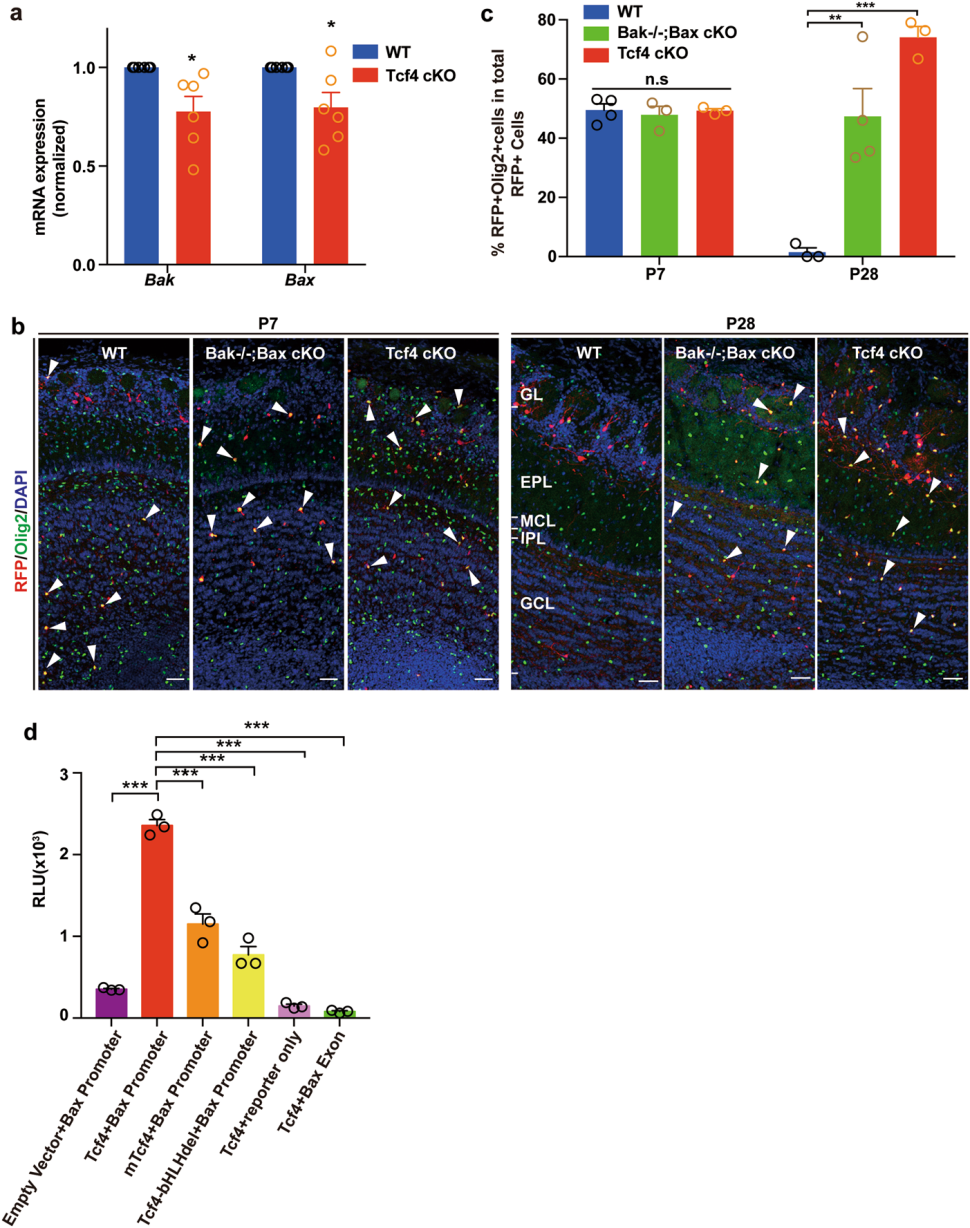
Tcf4 regulates OPC survival through Bax/Bak pathway. **a** Relative expression level of *Bax* and *Bak* in WT and Tcf4 cKO OBs. The expression level of WT was normalized to 1. Student’s *t* test was used to compare two groups. Data are presented as mean ± SEM. *p < 005. Six OBs were analyzed for each genotype. **b** Representative images show immunostaining for Olig2 and RFP at P7 and P28 in OBs of WT, Bak1−/−;Bax cKO and Tcf4 cKO. Arrowheads indicate OPCs. Scale bar: 50 μm. **c** Quantification of the number of OPCs from (**b**). Two-way ANOVA was used for statistical analysis. Data are presented as mean ± SEM. **p < 0.01, ***p < 0.001, n.s. nonsignificant. At least three OBs were analyzed for each genotype at each time point. **d** Tcf4 regulates Bax transcription examined by dual-luciferase assay. Tcf4 with disease-related point-mutations (mTcf4) and Tcf4 lacking the bHLH domain (Tcf4-bHLHdel) were used for examining the function of Tcf4 bHLH domain. Data are shown as mean ± SEM. One-way ANOVA, ***p < 0.001.

The pro-apoptotic Bcl2 family members Bcl2-associated X protein (Bax) and Bcl2 antagonist/killer 1 (Bak) are key regulators of the apoptosis signaling pathway^35,36^ and play essential roles in regulating neuronal cell death^8^. We reasoned that Tcf4 could potentially regulate Bax/Bak pathway to control OPC survival. To test this hypothesis, we first examined the expression of *Bax* and *Bak* in Tcf4 cKO OBs by real-time quantitative polymerase chain reaction (qPCR). We found that the mRNA expression level of *Bax* and *Bak* was reduced in Tcf4 cKO OBs compared to that of WT OBs (Fig. 7a). To further examine whether Tcf4 acts though Bax/Bak to control the OPC survival, we generated conditional knockout mice in which *Bax* and *Bak* were removed in Nkx2.1 lineage specifically by crossing Bak^−/−^;Bax^f/f^;Ai14 mice with Nkx2.1Cre line (Bak^−/−^;Bax cKO). At P7, the number of OPCs was similar in the OBs of WT, Bak^−/−^;Bax cKO and Tcf4 cKO (Fig. 7b, c). However, we found a significant increase of OPC when Bak and Bax were removed in the OB of Bak^−/−^;Bax cKO (Fig. 7b, c). Therefore, the suppression of cell death in Nkx2.1 lineage resulted in an increase of OPCs in the OB. We then performed a dual-luciferase transcriptional activation assay to examine whether Tcf4 regulates Bax expression (Fig. 7d). Indeed, we found that Tcf4 activated the transcription of the Bax promoter while no transcription activation was observed when Bax promoter was replaced with a fragment of its exon region (Fig. 7d). The activation of Bax transcription was dependent on the bHLH domain of Tcf4 as the transcription activation was abolished when the bHLH domain of Tcf4 was removed (Fig. 7d, Tcf4-bHLHdel). Interestingly, when Tcf4 carried point mutations at R578H, R580W, and R582P within the bHLH domain, which were found in patients with Pitt–Hopkins syndrome^37^, the transcription activation of Bax was reduced significantly (Fig. 7d, mTcf4). Taken together, Bax/Bak pathway is a potential key downstream target of Tcf4 to control OPC survival.

## Discussion

**Fig. 8:**
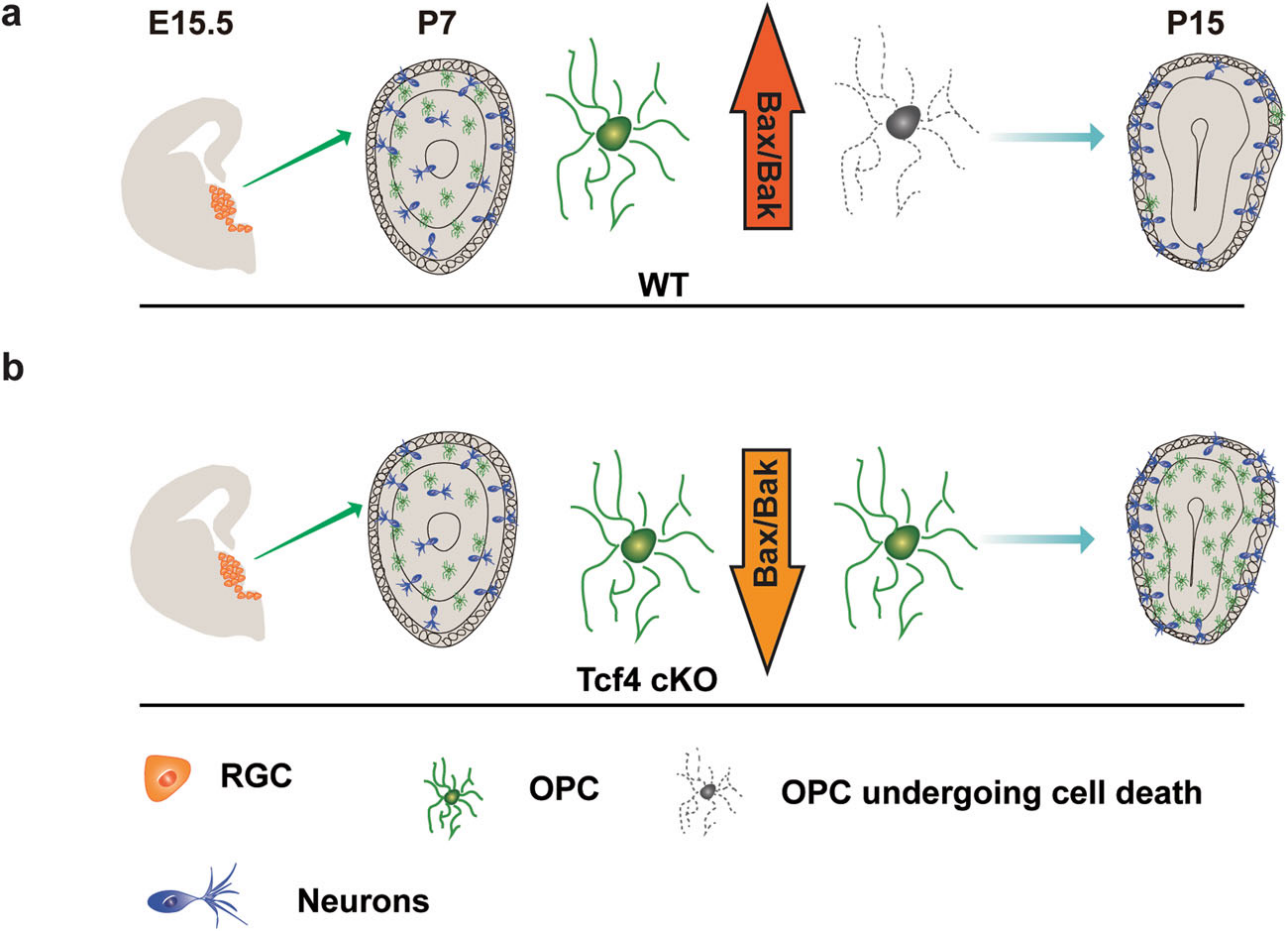
A model that illustrates how the OPC number is controlled by Tcf4 mediated cell death. **a** MGE- and AEP-derived OPCs undergo cell death through Bax/Bak pathway after the first postnatal week. **b** However, when Tcf4 is removed, the downregulation of Bax/Bak leads to an increase of OPC survival.

In this study, we revealed a novel role for Tcf4 in regulating OPC survival during OB development (Fig. 8). We showed that Nkx2.1-positive RGCs contribute to the first wave of OPCs in the OB. When Tcf4 was conditionally removed in the Nkx2.1 lineage, the number of OPCs in the OB was significantly increased, which was not due to the enhanced OPC differentiation. Unexpectedly, we found that Tcf4 acts on Bax/Bak pathway to control the OPC survival during OB development. The increased OPCs in the OB could potentially impact neuronal functions, which provides insights into TCF4-related brain disorders.

Dysregulation of oligodendrocyte production has been implicated in the disruption of axonal myelination, which leads to severe neurological disorders^38,39^. Particularly, impairments in olfaction are often associated with autism spectrum disorders^40,41^, highlighting the importance of neural circuits in the olfactory system. Oligodendrocyte mediated myelination could play essential roles in regulating neuronal functions in the OB. Indeed, disruption of OPC differentiation toward oligodendrocytes has been found in CHD7/CHD8 mutations, which result in CHARGE syndrome and autism spectrum disorders^42^. Therefore, tight control of the number of OPCs, thereby oligodendrocytes in the OB is important for myelination related disorders. We found that programmed cell death plays a pivotal role in maintaining a precise number of OPCs during OB development, which could be an essential survival control of OPCs and potentially guarantee proper olfactory functions.

TCF4 mutations cause Pitt-Hopkins syndrome, a rare neurodevelopmental disorder characterized by intellectual disability and autistic behavior^22,28,43^. In addition, genetic variants in TCF4 have been linked to neuropsychiatric diseases, for example, schizophrenia and autism^20,28,44^. Loss of Tcf4 in neurons leads to alteration of neuronal activity^26^ and deficits in learning and memory^27^. We show here that Tcf4 plays an essential role in OPC development. The proper number of OPC is crucial for oligodendrocyte maturation which is fundamental for axonal myelination. For example, dysregulation of oligodendrocyte development impairs the cognitive function in mouse models^45^. Therefore, Tcf4-mediated OPC survival may underlie the neurological deficits related to TCF4 mutations. Indeed, disruption of oligodendrocyte development has been implicated as a common feature shared by Pitt–Hopkins syndrome and autism spectrum disorder^28^. Therefore, our studies revealed the importance of Tcf4 in controlling OPC survival during OB development, which provides novel insights into the mechanisms underlying TCF4-associated brain disorders.

## Material and methods

### Animals

All animal work was approved by the Animal Care and Use Committee of Shanghai Medical College of Fudan University. The following mice were used: Tcf4^flox/flox^ mice^22^, Nkx2.1-Cre mice^14^, Bak^−/−^;Bax^flox/flox^ mice^46^, Ai14 or Ai3 reporter mice. Animals were bred under the condition of a 12-h light/dark cycle.

### Immunohistochemistry

Deeply anesthetized neonatal mice were transcardially perfused with phosphate-buffered saline (PBS) followed by 4% paraformaldehyde (PFA). Then brains were then post-fixed overnight at 4 °C. Embryonic brains were harvested and fixed in 4% PFA overnight at 4 °C. After incubation with blocking buffer (0.05% Triton X-100, 5% normal donkey serum in PBS), cryosections were incubated with desired antibodies overnight at 4 °C and then with appropriate fluorescence-conjugated secondary antibodies at room temperature for 1 h before mounting.

### Image acquisition and statistical analysis

Confocal images were acquired using Nikon A1R confocal microscope. Images were processed and analyzed using NIS—Elements AR (Nikon) and ImageJ software. For data analysis, Prism7 or Excel was used. All data are presented as mean ± SEM. Significances are marked as *p < 0.05, **p < 0.01, ***p < 0.001. Unless specified, two comparisons were tested using two-tailed unpaired Student’s *t* tests. Multiple comparisons were analyzed using ANOVA.

### Flow cytometry analysis

Neural tissue dissociation kit (P) (Miltenyi Biotec;130–093-231) was used to dissociate the OB from Tcf4 cKO and WT mice at P7. The cell suspension was incubated with antibody anti-PDGFRα (1:500, 558774, BD-Bioscience), followed by the incubation with anti-rat with Alexa647 (1:600, 712-605-153, Jackson ImmunoResearch) in dark for 30 min on ice. Cells were then incubated with Annexin V-FITC (640945, Biolegend) for 20 min at 37 °C. After washing, cells were analyzed by flow cytometry (MoFloAstrios EQ) according to the manufacturer’s instructions.

### Transplantation

Embryonic MGE and AEP (E15.5) were harvested from both WT and Tcf4 cKO mice. After dissociation, a single-cell suspension was injected into the OBs of WT host mice at P1 guided by stereotaxic apparatus (position: anteroposterior to bregma (AP) 1.3 mm, lateral to midline (L) 0.6 mm, ventral to the dura (V) 0.5 mm) according to a protocol described previously^47^.

### Tamoxifen and BrdU injection

Tamoxifen (Sigma, T5648) was dissolved in corn oil to prepare the stock solution at a concentration of 20 mg/ml. Tamoxifen was injected intraperitoneally with a dose of 10 µg/kg body weight. BrdU (Sigma, B5002) was dissolved in PBS to prepare the stock solution at the concentration of 10 mg/ml. BrdU was injected intraperitoneally at a concentration of 0.1 μg/kg body weight.

### Dual-luciferase assay

The *Bax* promoter was amplified by PCR and cloned into luciferase reporter pGL4.0 vectors (Promega). Point mutations of *hTCF4* (human *TCF4*) were made through PCR amplification. Gene sequences were confirmed by Sanger sequencing. The Dual-Luciferase Reporter Assay System (Promega) was performed according to the manufacturer’s instructions.

## Supplementary information

Figure S1

Figure S2

Supplemental Figure legends
