# Supplementary figures and images for: Survival control of oligodendrocyte progenitor cells requires the transcription factor 4 during olfactory bulb development
Source: Cell Death Dis. 2021 Jan 18;12(1):91. doi: 10.1038/s41419-020-03371-3 (PMC7813844; doi:10.1038/s41419-020-03371-3)

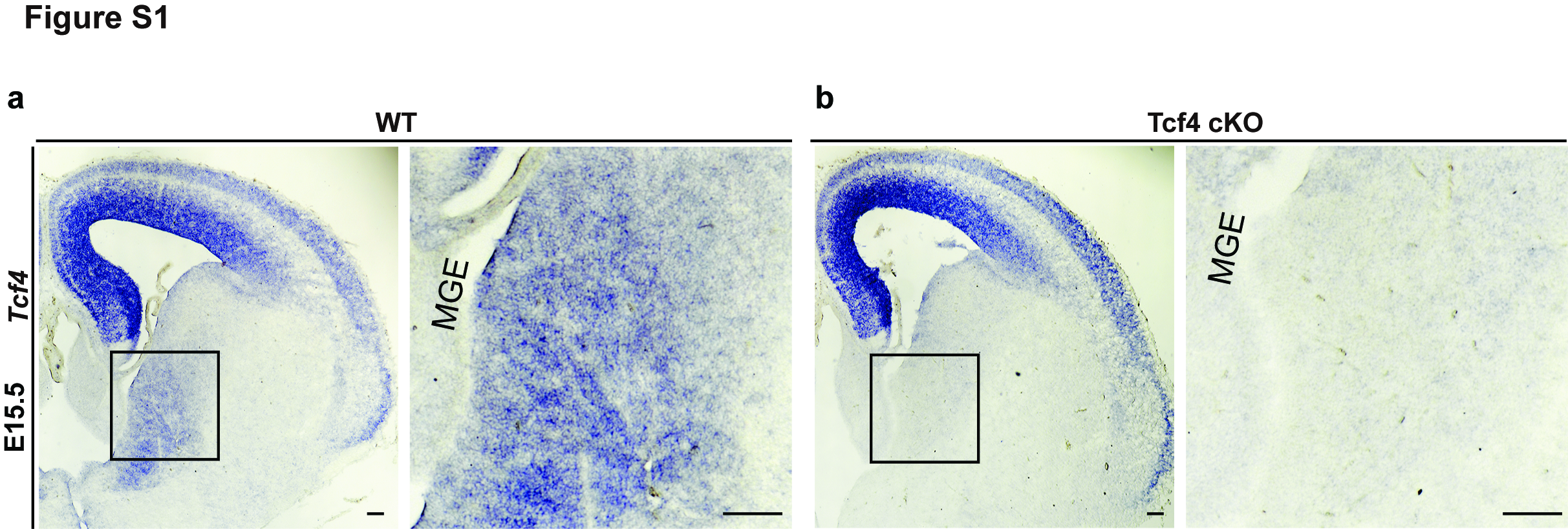

Supplement: Supplementary file 1 — Figure S1 [file 41419_2020_3371_MOESM1_ESM.tif]

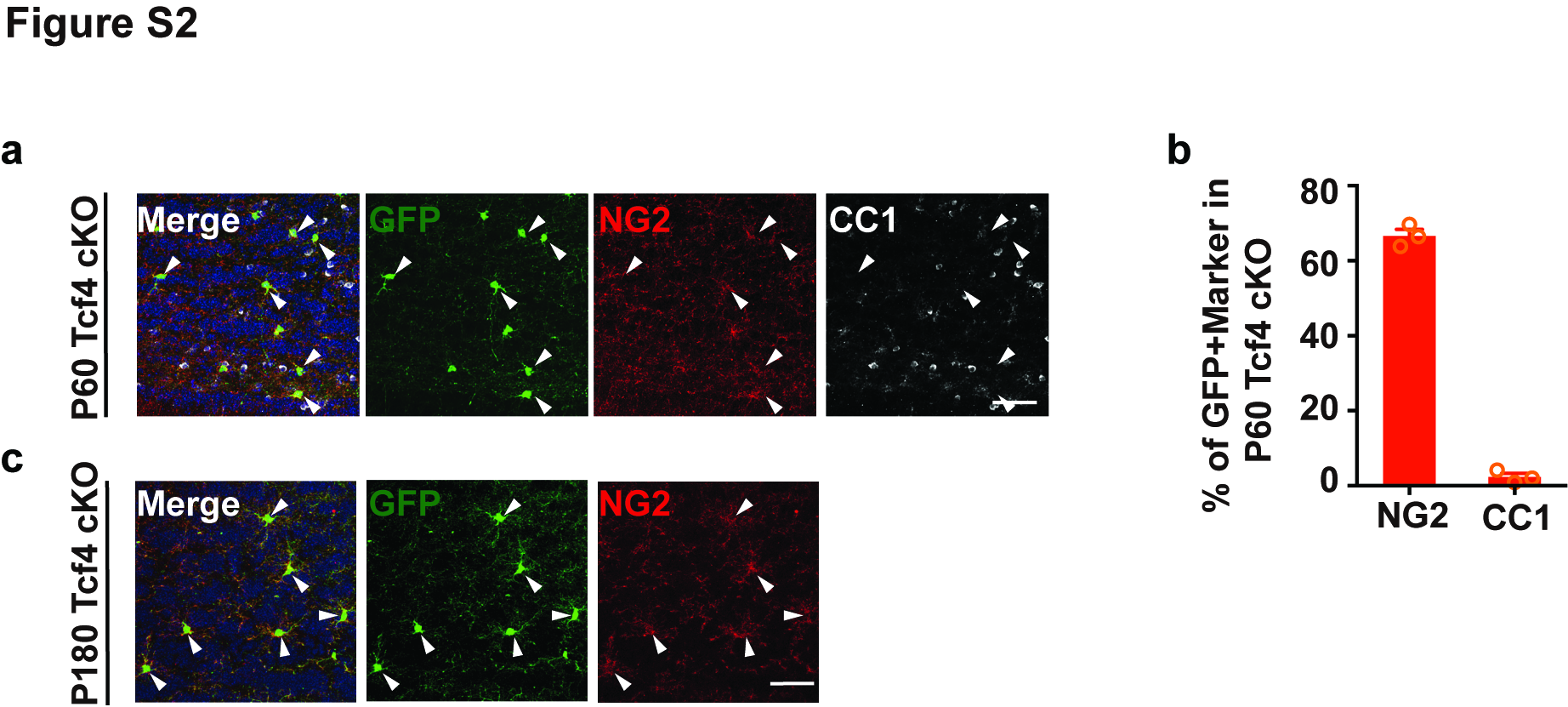

Supplement: Supplementary file 2 — Figure S2 [file 41419_2020_3371_MOESM2_ESM.tif]
