# Supplementary material for: Survival control of oligodendrocyte progenitor cells requires the transcription factor 4 during olfactory bulb development
Source: Cell Death Dis. 2021 Jan 18;12(1):91. doi: 10.1038/s41419-020-03371-3 (PMC7813844; doi:10.1038/s41419-020-03371-3)
Supplement: Supplementary file 3 — Supplemental Figure legends [file 41419_2020_3371_MOESM3_ESM.docx]

**SUPPLEMENTAL FIGURE LEGENDS**

**Fig. S1 Confirmation of Tcf4 depletion upon the expression of Nkx2.1Cre a** Images show Tcf4 expression in WT brain. **b** Upon the activation of Nkx2.1Cre, Tcf4 expression was specifically removed in MGE and AEP. Scale bar: 100μm.

**Fig. S2 Ectopic OPCs lacing Tcf4 persist till the adulthood a** Representative images show that OPCs in OBs of Tcf4 cKO are positive for OPC markers NG2, but not CC1. Scale bar: 50μm. **b** Quantification of the number of OPCs at P60. Data are shown as mean ± SEM. n = 3 mice for each timepoint. **c** Representative images show that OPCs persist in P180 mice. Scale bar: 50μm.
